# Grading of Neuroendocrine Carcinomas: Correlation of ^68^Ga-PET/CT Scan with Tissue Biomarkers

**DOI:** 10.1155/2018/6878409

**Published:** 2018-12-02

**Authors:** Chiara Liverani, Alberto Bongiovanni, Laura Mercatali, Flavia Foca, Federica Pieri, Alessandro De Vita, Chiara Spadazzi, Giacomo Miserocchi, Federica Recine, Nada Riva, Silvia Nicolini, Stefano Severi, Giovanni Martinelli, Toni Ibrahim

**Affiliations:** ^1^Osteoncology and Rare Tumors Center, Istituto Scientifico Romagnolo per lo Studio e la Cura dei Tumori (IRST) IRCCS, Meldola, Italy; ^2^Unit of Biostatistics and Clinical Trials, Istituto Scientifico Romagnolo per lo Studio e la Cura dei Tumori (IRST) IRCCS, Meldola, Italy; ^3^Nuclear Medicine Unit, Istituto Scientifico Romagnolo per lo Studio e la Cura dei Tumori (IRST) IRCCS, Meldola, Italy; ^4^Scientific Directorate, Istituto Scientifico Romagnolo per lo Studio e la Cura dei Tumori (IRST) IRCCS, Meldola, Italy

## Abstract

There is a growing need for more accurate biomarkers to facilitate the diagnosis and prognosis of patients with grade (G) 3 neuroendocrine carcinomas (NECs). In particular, the discrimination between well-differentiated neuroendocrine carcinomas (WD-NECs) and poorly differentiated neuroendocrine carcinomas (PD-NECs) is still an unmet need. We previously showed that ^68^Gallium-(^68^Ga-) PET/CT positivity is a prognostic factor in patients with gastroenteropancreatic (GEP) G3 NECs, correlating with a better outcome in terms of overall survival. Here, we hypothesize that ^68^Ga-PET/CT could help to discriminate between WD-NECs and PD-NECs, adding complementary information to that obtained from morphologic and biologic factors. A retrospective, single-institution study was performed on 11 patients with histologically confirmed, measurable G3 large- or small-cell GEP-NECs according to the 2017 WHO classification. The staging procedures included a ^68^Ga-PET/CT scan. Results of ^68^Ga-PET/CT were correlated in univariate analysis with loss of tissue immunohistochemical expression of DAXX/ATRX or RB1 frequently associated with WD-NECs or PD-NECs, respectively. None of the patients with positive ^68^Ga-PET/CT showed loss of RB1 expression, whereas among those (*n* = 6) with negative ^68^Ga-PET/CT, 4 showed loss of expression. A trend towards a correlation between loss of RB1 expression and negative ^68^Ga-PET/CT was observed. Our preliminary data support the hypothesis that PD-NECs carrying RB1 mutation and loss of its expression may be associated with negative ^68^Ga-PET/CT. If confirmed in a larger clinical trial, ^68^Ga-PET/CT would help in the stratification of G3 NECs.

## 1. Introduction

Poorly differentiated grade 3 (PD G3) gastroenteropancreatic (GEP) neuroendocrine carcinomas (NECs) are very rare malignancies that represent only 5%-10% of all neuroendocrine neoplasms (NENs) [1, 2]. These tumors are characterized by aggressive histological features such as high Ki67 index, extensive necrosis, and nuclear atypia [2]. At the time of diagnosis, patients are generally in poor conditions, with aggressive and diffuse disease [3, 4]. Due to the rarity of NECs, few dedicated prospective clinical or biological trials have been conducted. Furthermore, current NEC grading shows a number of controversies and discrepancies that highlight the need for more accurate biomarkers [5–9]. The revised 2010 World Health Organization (WHO) classification identified all GEP neuroendocrine tumors with Ki67 > 20% as grade 3 NECs [10]. Recent studies have shown that these tumors might actually include 2 heterogeneous subgroups with a different pathogenesis: well-differentiated neuroendocrine carcinomas (WD-NECs) characterized by mutations in *MEN1*, *DAXX*, and *ATRX* genes and poorly differentiated neuroendocrine carcinomas (PD-NECs) characterized by p53 and RB1 mutations probably derived from the neuroendocrine differentiation of adenocarcinomas [8, 11, 12]. There is evidence that these 2 subgroups also have a distinct prognosis and show different sensitivities to chemotherapy [3, 13]. A subdivision of tumors with Ki67 > 20% into G3 WD-NETs or G3 PD-NECs was proposed in the 2017 WHO classification for neuroendocrine neoplasms of pancreatic origin [14], leading to the identification of a new category comprising WD tumor morphology and Ki67 index > 20%, referred to as G3 pNETs. According to this classification, tumor grading is based on histopathologic morphology and on the assessment of the Ki67 index [15]. However, distinguishing G3 NETs from G3 NECs is often problematic due to the lack of well-defined histological criteria and differences in Ki67 assessment [16]. Moreover, the classification of NECs of different sites of origin has yet to be revised. According to international guidelines, the identification and evaluation of novel biomarkers is warranted. Tang et al. [8] reported that the 2 subgroups show a different positivity to ^18^F-fluorodeoxyglucose positron emission tomography/computerized tomography (^18^F-FDG-PET/CT) or octreoscan. In a recent study, we showed that ^68^Gallium- (^68^Ga-) PET/CT was a discriminating factor for patients with G3 GEP-NECs treated with first-line platinum-based chemotherapy. Patients with a positive ^68^Ga-PET/CT scan had a better outcome than those with a negative ^68^Ga-PET/CT (75% *vs.* 34.3% overall survival at 18 months, respectively) [17]. The identification of specific metabolic characteristics may be particularly useful when histological material is not available, and imaging studies could add complementary information to that obtained from morphologic and biologic factors. ^68^Gallium directly binds to somatostatin receptors (SSTRs) which are often overexpressed in the cell membrane of NENs, especially in WD tumors [18]. We hypothesized that ^68^Ga-PET/CT, reflecting the degree of neuroendocrine differentiation [19, 20], could help to distinguish between WD-NECs and PD-NECs. We conducted a preliminary study to assess whether ^68^Ga-PET/CT correlates with the specific mutations identified in the 2 subgroups, DAXX and ATRX for WD-NECs and RB1 for PD-NECs. Given that mutations in these genes are closely correlated with loss of immunolabeling [21, 22], we evaluated the tissue immunohistochemical expression of DAXX, ATRX, and RB1 in 11 patients with G3 GEP-NECs. We then compared the expression of these markers with results of ^68^Ga-PET/CT to look for potential correlations with metabolic parameters that could help to discriminate between WD-NECs and PD-NECs.

## 2. Materials and Methods

### 2.1. Study Design

We retrospectively evaluated 11 patients seen at our institute (IRST IRCCS, Meldola) between April 2010 and May 2018. The patients were required to have histologically confirmed, measurable G3 large- or small-cell GEP-origin NECs. All cases were revised by an expert pathologist and divided into poorly differentiated G3 NECs or well-differentiated G3 NETs according to the 2017 WHO classification for pancreatic NENs, as reported by Sorbye et al. [23]. Patients with mixed tumors were excluded. The study was reviewed and approved by IRST IRCCS Medical Scientific Committee and Ethics Committee. Staging procedures performed included physical examination, brain-chest-abdominal CT, and ^68^Ga- and FDG-PET/CT.

### 2.2. Immunohistochemical Analysis

Paraffin-embedded surgical or biopsy specimens of G3 neuroendocrine tumors were sliced with a rotating microtome (Leica Biosystems, Wetzlar, Germany) into 5 *μ*M thick sections and mounted on SuperFrost Plus microslides (Thermo Fisher Scientific, Waltman, MA, USA). Immunolabeling reactions were carried out on a VENTANA BenchMark XT (Ventana Medical Systems Inc., Tucson, AZ, USA) automated slide strainer. The following antibodies were used according to the manufacturer's instructions: DAXX (HPA008736) (Sigma-Aldrich, St. Louis, MO, USA) 1 : 75, one hour at room temperature (RT); ATRX (HPA001906) (Sigma-Aldrich) 1 : 400, one hour at RT; and RB1 (Cell Signaling Technology, Beverly, Massachusetts, USA) 1 : 1000, one hour at RT. The stained sections were analyzed in blind by an expert pathologist in neuroendocrine neoplasms.

### 2.3. Imaging with ^68^Ga-Labeled Somatostatin Analogs

^68^Ga-labeled somatostatin analogs are generally short peptides linked to the positron emitter ^68^Ga by a bifunctional chelate, normally 1,4,7,10-tetraazacyclododecane-1,4,7,10-tetraacetic acid (DOTA). ^68^Ga-DOTA peptides bind to SSTRs, in particular SSTR3 and SSTR5, both of which are usually overexpressed in neuroendocrine cells [24]. There are 3 main ^68^Ga-DOTA-peptides currently available for imaging procedures on the basis of their affinity for SSTR subtypes. We used ^68^Ga-DOTA-Phe1-Tyr3-octreotide (TOC), which has a high affinity for SSTR2 and SSTR5 [25].

### 2.4. Statistical Analysis

Continuous variables were expressed as mean and standard deviation (SD), while categorical variables were expressed as frequency. Fisher's exact test was used to evaluate the relationship between categorical variables. Median overall survival (OS) was estimated as an exploratory research objective using the Kaplan-Meier method (two-sided 95% confidence intervals (CIs)). Reported *P* values <0.05 were used as a threshold for significance. Statistical analyses were carried out with STATA/MP 10.1 for Windows (StataCorp LP, College Station, TX, USA).

## 3. Results

### 3.1. Clinical Features

The main clinical and histological characteristics of the 11 patients analyzed in this study are shown in Table 1. Six patients (54.5%) were males and 5 (45.5%) were females. Mean age at the time of diagnosis was 56.6 years (SD ± 13.1). The site of the primary tumor was pancreas in 5 patients (45.6%), stomach in 3 (27.2%), and colorectum in 3 (27.2%). Six patients (54.5%) had well-differentiated G3 NETs and 5 (45.5%) poorly differentiated NECs. Patients received first-line chemotherapy with platinum compounds and etoposide (8 with cisplatin and 2 with carboplatin). Of the 11 patients, 5 (45.6%) showed a partial response (PR), 3 (27.2%) stable disease (SD), and 3 (27.2%) progressive disease (PD). Median follow-up was 32 months (range 5-86). Median OS was 23 months (95% CI: 7-70). No difference in survival was observed in relation to gender or age (data not shown). Four patients had a positive ^68^Ga-PET/CT and 6 a negative ^68^Ga-PET/CT, while the ^68^Ga-PET/CT or octreoscan referral for the remaining patient was not available.

### 3.2. DAXX, ATRX, and RB1 Immunohistochemical Expression

**Figure 1:**
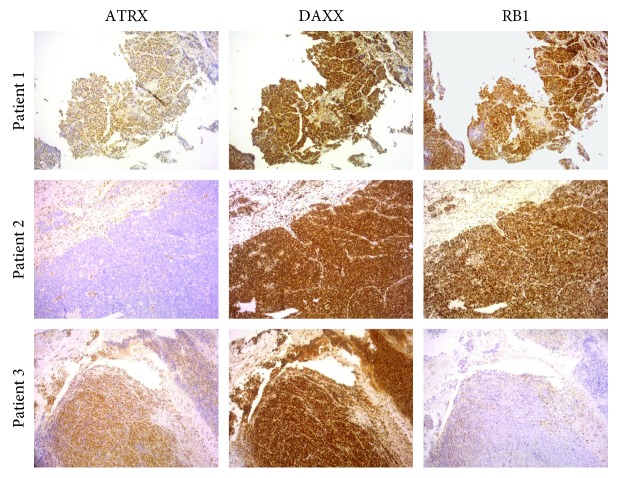
NEC tissue immunostained for ATRX, DAXX, and RB1. Patient 1 showed positive immunostaining of all 3 markers. Patient 2 showed positive expression of DAXX and RB1 and loss of ATRX expression. Patient 3 showed positive expression of ATRX and DAXX and loss of RB1 expression. Magnification ×10.

Expression of DAXX, ATRX, and RB1 in G3 neuroendocrine tumor tissue is shown in Table 2. All markers showed a strong nuclear localization, and stromal cells were used as an internal positive control for immunostaining (Figure 1). It was not possible to evaluate the expression of the three biomarkers in 2 patients due to insufficient bioptic material. DAXX was expressed in 100% of neuroendocrine tumor tissue, and no patient showed loss of IHC expression of this marker. ATRX was expressed in 66.7% of neuroendocrine tumor tissues, and 3 (33.3%) patients showed a loss of expression. Interestingly, all patients with loss of ATRX expression had NECs of gastrointestinal origin. DAXX and ATRX mutations are mutually exclusive. RB1 was expressed in 44.5% of neuroendocrine tumor tissue, and 5 (55.5%) patients showed a loss of expression. Of these, one had pancreatic NEC and 4 had gastrointestinal NECs.

### 3.3. Correlation between DAXX/ATRX and RB1 Expression and ^68^Ga-PET/CT

The correlation between DAXX/ATRX or RB1 expression and ^68^Ga-PET/CT scan is reported in Table 3. Bioptic material was not evaluable in 2 patients with a positive ^68^Ga-PET/CT. The other 2 ^68^Ga-PET/CT-positive patients showed expression of ATRX/DAXX. Of the 6 patients with negative ^68^Ga-PET/CT, 4 showed ATRX/DAXX expression and 2 patients showed a loss of expression. With regard to RB1, patients with positive ^68^Ga-PET/CT showed expression of this marker. Among those with negative ^68^Ga-PET/CT, 2 showed RB1 expression and 4 patients a loss of expression.

### 3.4. Correlation between Histological Grading and ^68^Ga-PET/CT

The correlation between histological grading and ^68^Ga-PET/CT scan is reported in Table 4. Of the 4 patients with positive ^68^Ga-PET/CT, 3 had G3 NETs and one had G3 NEC. Of the 6 patients with negative ^68^Ga-PET/CT, 2 had G3 NETs and 4 had G3 NECs.

### 3.5. Correlation between ATRX/DAXX and RB1 Expression, ^68^Ga-PET/CT, and Histological Grading and Best Response to First-Line Chemotherapy

The correlation between ATRX/DAXX or RB1 expression and best response to first-line chemotherapy is shown in Table 5. Of the 6 patients showing ATRX/DAXX expression, 3 had PR or SD and 3 PD. All 3 patients showing a loss of ATRX/DAXX expression had PR or SD, which is consistent with the less aggressive behavior of WD tumors. Of the 4 patients showing RB1 expression, 2 had PR or SD and 2 showed PD. Of the 5 patients with loss of RB1, 4 showed PR or SD and one PD. The correlation between ^68^Ga-PET/CT and best response to first-line chemotherapy is reported in Table 6. Of the 6 patients with negative ^68^Ga-PET/CT, 4 had PR or SD and 2 had PD. Of the 4 patients with positive ^68^Ga-PET/CT, 3 showed PR or SD and one had PD. The correlation between histological classification and best response to first-line chemotherapy is reported in Table 7. Of the 6 patients with G3 NETs, 4 had PR or SD and 2 showed PD. Of the 5 with G3 NECs, 4 had PR or SD and one had PD.

### 3.6. PFS and OS according to DAXX/ATRX or RB1 Expression

The median PFS (mPFS) and OS (mOS) of the different subgroups on the basis of DAXX/ATRX and RB1 expression is shown in Table 8. mPFS was 6 months in the group with DAXX/ATRX-negative tumors and 3 months in those with DAXX/ATRX-positive disease. mPFS was 7 months in patients with RB1-negative tumors and 3 months in those with RB1-positive disease. mOS was 6 months in the group with DAXX/ATRX-negative tumors and 11 months in those with DAXX/ATRX-positive disease. mOS was 11 months in the group with RB1-negative tumors and 6 months for those with RB1 positivity.

## 4. Discussion

The updated 2017 WHO tumor classification divided pancreatic neuroendocrine carcinomas into G3 NETs characterized by Ki67 > 20% and a well-differentiated morphology, or G3 NECs characterized by Ki67%>20% and the absence of a low-grade component [14]. Given the lack of objective and well-defined histological criteria and consensus on Ki67 evaluation, more accurate biomarkers are needed. The authors of the NORDIC NEC study showed that G3 NEC patients with Ki67 > 55% were more sensitive to platinum-based chemotherapy but had poorer survival [3]. However, the study did not consider histopathological characteristics. Other trials have evaluated the diagnostic, prognostic, or predictive value of different biological markers such as serum plasma [26] and tissue [27] markers. Metabolic analysis plays an important role in the management of NEC patients in terms of diagnosis, staging, and therapeutic decision-making [28, 29]. In particular, ^68^Ga-PET/CT imaging provides information on SSTR expression [30], while ^18^F-FDG-PET/CT defines tumor metabolic status [31]. Although ^18^F-FDG-PET/CT has shown limited value in WD-NETs as they seldom show alterations in glucose turnover [32], the technique has emerged as a promising marker of aggressiveness and metastasis.

In the present study, we evaluated the potential correlation between ^68^Ga-PET/CT and loss of expression of tissue biomarkers specific for WD-NECs and PD-NECs in an attempt to define the value of ^68^Ga-PET/CT in discriminating between the 2 subgroups. In particular, we observed that none of the ^68^Ga-PET/CT-positive patients showed loss of RB1 expression, whereas among those with negative ^68^Ga-PET/CT, 4 (66.7%) showed loss of expression of RB1 and 2 (33.3%) normal expression. A trend towards a correlation between negative ^68^Ga-PET/CT and loss of RB1 expression emerged. Moreover, there was good agreement between ^68^Ga-PET/CT results and histological classification according to the 2017 WHO classification. Specifically, of the 4 patients with positive ^68^Ga-PET/CT, 3 (75.5%) had G3 NETs, while of the 6 patients with negative ^68^Ga-PET/CT, 4 (66.7%) had G3 NECs. These preliminary data support the hypothesis that negative ^68^Ga-PET/CT, reflecting a lower degree of neuroendocrine differentiation [19, 20], may be associated with the PD-NEC subgroup that frequently harbors an RB1 mutation and loss of its expression [8]. If confirmed in larger clinical trials, ^68^Ga-PET/CT could provide important complementary information to facilitate G3 NEC stratification. Given that NEC patients often present with metastatic disease and that histological material may thus not be available, metabolic imaging could substitute histological analysis in such cases. Further research is also needed to assess the impact of the proposed stratification on the definition of disease outcome in terms of PFS, OS, and response to therapy. The use of imaging analysis for the grading and prognosis assessment of NEN patients has been investigated by other groups. In a recent study by Zhao et al., pharmacokinetic parameters of dynamic contrast-enhanced magnetic resonance imaging were found to be predictive of NET grading, helping to distinguish between G1 and G2 tumors [33]. Another study reported that CT texture analysis and CT features were predictive of pancreatic NET aggressiveness and could be used to identify patients at risk of early disease progression after surgical resection [34]. We thus believe that in-depth research is warranted to investigate the role of radiologic and metabolic imaging as diagnostic, prognostic, or predictive tools in NEN patients.

In conclusion, the results from the present study show the potential value of investigating ^68^Ga-PET/CT as a marker to distinguish between WD-NETs and PD-NECs. Confirmation of our findings in larger case series, ideally in multicenter and prospective settings, would help to better define NEC patient prognosis and predict response to treatment.

## Figures and Tables

**Table 1:** Clinical and histological characteristics of NEC patient samples.

	*n* (%)
Age at diagnosis (years)	
Mean	56.6
Standard deviation	13.1
Gender	
Male	6 (54.5)
Female	5 (45.5)
Site of disease	
Stomach	3 (27.2)
Colorectum	3 (27.2)
Pancreas	5 (45.6)
Histological classification	
G3 NET	6 (54.5)
G3 NEC	5 (45.5)
FDG-PET/CT	
Positive	8 (72.7)
Not done	3 (27.3)
^68^Ga-PET/CT octreoscan	
Positive	4 (36.4)
Negative	6 (54.5)
Not done	1 (9.1)
Chemotherapy	
CDDP	8 (72.7)
Carboplatin	2 (18.1)
Other	1 (9.2)
Best response to first-line chemotherapy	
PD	3 (27.2)
SD	3 (27.2)
PR	5 (45.6)
Median overall survival, months [range]	23 [6-70]

NEC: neuroendocrine carcinoma; NET: neuroendocrine tumor; G: grade; FDG-PET/CT: fluorodeoxyglucose-positron emission tomography/computerized tomography; ^68^Ga: Gallium-68; CDDP: cisplatin; PD: progressive disease; SD: stable disease; PR: partial response.

**Table 2:** Immunohistochemical expression of ATRX, DAXX, and RB1.

	Total (%)	Pancreatic (%)	GI (%)
ATRX			
Positive	6 (66.7)	3 (100.0)	3 (50.0)
Negative	3 (33.3)	0 (0.0)	3 (50.0)
Not evaluated	2	2	—
DAXX			
Positive	9 (100)	3 (100.0)	6 (100.0)
Negative	0 (0)	0 (0.0)	0 (0.0)
Not evaluated	2	2	—
ATRX + DAXX			
Positive	6 (66.7)	3 (100.0)	3 (50.0)
Negative	3 (33.3)	0 (0.0)	3 (50.0)
Not evaluated	2	2	—
RB1			
Positive	4 (44.5)	2 (66.7)	2 (33.3)
Negative	5 (55.5)	1 (33.3)	4 (66.7)
Not evaluated	2	2	—

GI: gastrointestinal.

**Table 3:** Correlation between ATRX + DAXX and RB1 expression and ^68^Ga-PET/CT or octreoscan positivity.

	^68^Ga-PET/CT or octreoscan results						
	Overall			Pancreatic		GI	
	Negative (%)	Positive (%)	*P* value^∗^	Negative (%)	Positive (%)	Negative (%)	Positive (%)
Overall	6 (66.7)	2 (33.3)		1 (33.3)	2 (66.7)	5 (100.0)	0 (0.0)
ATRX + DAXX							
Negative	2 (33.3)	0 (0.0)	0.536	0 (0.0)	0 (0.0)	2 (40.0)	0 (0.0)
Positive	4 (66.7)	2 (100.0)		1 (100.0)	2 (100.0)	3 (60.0)	0 (0.0)
RB1							
Negative	4 (66.7)	0 (0.0)	0.214	1 (100.0)	0 (0.0)	3 (60.0)	0 (0.0)
Positive	2 (33.3)	2 (100.0)		0 (0.0)	2 (100.0)	2 (40.0)	0 (0.0)

GI: gastrointestinal. ^∗^*P*-value was calculated on the overall number of patients.

**Table 4:** Correlation between histological classification and ^68^Ga-PET/CT or octreoscan positivity.

	^68^Ga-PET/CT or octreoscan results						
	Overall		*P* value^∗^	Pancreatic		GI	
	Negative (%)	Positive (%)		Negative (%)	Positive (%)	Negative (%)	Positive (%)
Overall	6 (60.0)	4 (40.0)		1 (20.0)	4 (80.0)	5 (100.0)	0 (0.0)
Histological classification							
NET G3	2 (33.3)	3 (75.0)	0.524	0 (0.0)	3 (75.0)	2 (40.0)	0 (0.0)
NEC G3	4 (66.7)	1 (25.0)		1 (100.0)	1 (25.0)	3 (60.0)	0 (0.0)

NET: neuroendocrine tumor; NEC: neuroendocrine carcinoma; GI: gastrointestinal.

**Table 5:** Correlation between ATRX + DAXX and RB1 expression and best response to first-line chemotherapy.

	Best response						
	Overall		*P* value^∗^	Pancreatic		GI	
	PD (%)	SD + PR (%)		PD (%)	SD + PR (%)	PD (%)	SD + PR (%)
Overall	3 (33.3)	6 (66.7)		1 (33.3)	2 (66.7)	2 (33.3)	4 (66.7)
ATRX + DAXX							
Negative	0 (0.0)	3 (50.0)	0.464	0 (0.0)	0 (0.0)	0 (0.0)	3 (75.0)
Positive	3 (100.0)	3 (50.0)		1 (100.0)	2 (100.0)	2 (100.0)	1 (25.0)
RB1							
Negative	1 (33.3)	4 (66.7)	0.524	0 (0.0)	1 (50.0)	1 (50.0)	3 (75.0)
Positive	2 (66.7)	2 (33.3)		1 (100.0)	1 (50.0)	1 (50.0)	1 (25.0)

PD: progressive disease; SD: stable disease; PR: partial response; GI: gastrointestinal. ^∗^*P*-value was calculated on the overall number of patients.

**Table 6:** Correlation between ^68^Ga-PET/CT or octreoscan and best response to first-line chemotherapy.

	Best response						
	Overall		*P* value^∗^	Pancreatic		GI	
	PD (%)	SD + PR (%)		PD (%)	SD + PR (%)	PD (%)	SD + PR (%)
Overall	3 (30.0)	7 (70.0)		1 (20.0)	4 (80.0)	2 (40.0)	3 (60.0)
^68^Ga-PET/CT or octreoscan							
Negative	2 (66.7)	4 (57.1)	0.667	0 (0.0)	1 (25.0)	2 (100.0)	3 (100.0)
Positive	1 (33.3)	3 (42.9)		1 (100.0)	3 (75.0)	0 (0.0)	0 (0.0)

PD: progressive disease; SD: stable disease; PR: partial response; GI: gastrointestinal. ^∗^*P* value was calculated on the overall number of patients.

**Table 7:** Correlation between histological classification and best response to first-line chemotherapy.

	Best response						
	Overall		*P* value^∗^	Pancreatic		GI	
	PD (%)	SD + PR (%)		PD (%)	SD + PR (%)	PD (%)	SD + PR (%)
Overall	3 (27.3)	8 (72.7)		1 (20.0)	4 (80.0)	2 (33.3)	4 (66.7)
Histological classification							
NET G3	2 (66.7)	4 (50.0)	1.000	1 (100.0)	2 (50.0)	1 (50.0)	2 (50.0)
NEC G3	1 (33.3)	4 (50.0)		0 (0.0)	2 (50.0)	1 (50.0)	2 (50.0)

PD: progressive disease: SD: stable disease; PR: partial response; GI: gastrointestinal; NET: neuroendocrine tumor; NEC: neuroendocrine carcinoma. ^∗^*P*-value was calculated on the overall number of patients.

**Table 8:** PFS and OS according to ATRX + DAXX and RB1 expression.

	ATRX + DAXX	
	Negative (*n* = 3)	Positive (*n* = 6)
Median PFS (95% CI) (months)	6 (6-NE)	3 (2-NE)
Median OS (95% CI) (months)	6 (5-NE)	11 (5-NE)

	RB1	
	Negative (*n* = 5)	Positive (*n* = 4)
Median PFS (95% CI) (months)	7 (2-NE)	3 (3-NE)
Median OS (95% CI) (months)	11 (6-NE)	6 (5-NE)

PFS: progression-free survival; OS: overall survival; CI: confidence interval; NE: not estimated.

## Data Availability

The data used to support the findings of this study are available from the corresponding author upon request.
